# Noncoding RNAs as Biomarkers for Acute Coronary Syndrome

**DOI:** 10.1155/2020/3298696

**Published:** 2020-04-01

**Authors:** Lijie Wang, Yuanzhe Jin

**Affiliations:** Department of Cardiology, The Fourth Affiliated Hospital of China Medical University, Shenyang, Liaoning 110032, China

## Abstract

Acute coronary syndrome (ACS), consisting of acute myocardial infarction and unstable angina, is the most dangerous and fatal form of coronary heart disease. Acute coronary syndrome has sudden onset and rapid development, which may lead to malignant life-threatening conditions at any time. Therefore, early detection and diagnosis are critical for patients with ACS. Recent studies have found that noncoding RNA is of great significance in the diagnosis and treatment of cardiovascular diseases. In this review, we summarized recent data on circulating noncoding RNAs (including microRNA, long noncoding RNA, and circular RNA) as diagnostic and prognostic markers in ACS including acute myocardial infarction and unstable angina. Specifically, microRNAs (miRNAs) as diagnostic markers are divided into three types: miRNAs of increased expression in ACS, miRNAs of decreased expression in ACS, and miRNAs of contradictory expression in ACS. Moreover, we described these miRNAs of increased expression in ACS based on miRNAs family. This review may result in a great guidance of noncoding RNAs as biomarkers for ACS in clinical practice.

## 1. Introduction

Cardiovascular disease (CVD) is now the leading cause of death worldwide, based on the data from the World Health Organization (WHO). Cardiovascular disease accounts for over 4 million deaths per year in Europe, close to 45% of all deaths, of which coronary heart disease is responsible for 20% [1]. In 2015, more than 2.7 million resident deaths are attributable to CVD in the United States, approximately 44% of which account for coronary heart disease (CHD) [2]. In 2015, the mortality rate of CHD in mainland China was about 110/100,000, and it increased year by year [3].

Acute coronary syndrome (ACS) is the most dangerous and fatal form of coronary heart disease. In the United States, the number of patients discharged with ACS as the first diagnosis in 2014 was 633,000 [2], and the 30-day mortality rate of patients with ACS was 5.3% in males and 9.6% in females [4]. Acute coronary syndrome can be divided into ST-Segment Elevation acute coronary syndrome (STE-ACS) and Non-ST-Segment Elevation acute coronary syndrome (NSTE-ACS) according to the presence or absence of ST-segment elevation in electrocardiogram. The former refers to acute ST-Segment Elevation Myocardial Infarction (STEMI), and the pathological mechanism is coronary artery total occlusion. The latter refers to acute Non-ST-Segment Elevation Myocardial Infarction and unstable angina pectoris. The pathological mechanism may be the coronary artery noncomplete occlusion. Acute coronary syndrome has sudden onset and rapid development, which may lead to malignant life-threatening conditions at any time. Therefore, early detection and diagnosis are critical for patients with ACS.

Among the current clinical biomarkers commonly used in ACS, mainly including Cardiac troponin I (cTnI), Cardiac troponin T (cTnT), creatine kinase-MB (CK-MB), and myoglobin, cTnI/cTnT (especially the hypersensitive troponin I/T) is myocardial necrosis markers with higher sensitivity and specificity. However, the level of troponin I/T was also found increased in other diseases than myocardial ischemic necrosis recently [5–7]. Moreover, for unstable angina pectoris, the effective clinical biomarkers are still not available. Therefore, for patients with ACS (especially UA), it is particularly important to explore more specific, sensitive, and stable novel biomarkers.

Over 90% of the human genome is composed of noncoding RNAs (ncRNAs), which used to be considered as genetic junk. These noncoding RNAs have great effects on the posttranscriptional gene regulation and the development of many diseases. Currently, recognized ncRNAs refer to all RNAs that cannot be translated into proteins except rRNA and tRNA, mainly including microRNA (miRNA/miR), long noncoding RNA (lncRNA), and circular RNA (circRNA). miRNA is an ncRNA with a short single chain structure of only about 22 nucleotides. lncRNA is a long chain ncRNA with more than 200 nucleotide structures. circRNA is an endogenous ncRNA without 5′-3′ polarity and a long cyclic polyadenylate tail, which is mainly distributed in the nucleus. circRNAs are closed loop molecules composed of covalent closed loop structures that are resistant to RNA exonucleotide enzymes that only digest linear transcripts. Thus, circRNAs are more stable than linear RNAs. Furthermore, circRNAs are appropriate biomarkers for disease diagnosis based on their stability in blood and other body fluids. circRNAs can regulate gene expression and participate in various pathophysiological processes. Specific mechanisms include adsorption of certain miRNAs to prevent mRNA translation, binding RNA-related proteins to form functional complexes; as a transcriptional regulator, it directly affects gene expression.

All these three kinds of ncRNAs are directly or indirectly involved in the regulation of gene expression and the development of cardiovascular diseases. Recent studies have found that ncRNA is of great significance in the diagnosis and treatment of cardiovascular diseases [8]. This review reviews the progress of peripheral blood ncRNA as a diagnostic and prognostic marker in ACS.

## 2. Materials and Methods

For this review, we searched the PubMed/Medline database and selected published researches that investigate noncoding RNAs as biomarkers for acute coronary syndrome up to Aug 18, 2019. The Fourth Affiliated Hospital of China Medical University Clinical Research Ethics Committee approved the protocol of this study and that the approval number is EC-2019-KS-070. The search terms included “microRNA”, “LncRNA”, “circRNA”, “acute myocardial infarction”, “acute coronary syndrome”, “unstable angina”, “coronary artery disease”, and “biomarkers”.

The inclusion criteria were (1) a study population of humans, (2) a sample of peripheral blood/serum/plasma, (3) biomarkers for diagnosis and prognosis, and (4) regular detection by RT-PCT/real-time PCR/Microarray.

The exclusion criteria were (1) a study population of animals, (2) a sample of other than peripheral blood/serum/plasma, (3) biomarkers of other than diagnosis and prognosis, (4) detections of other than RT-PCT/real-time PCR/Microarray, (5) patients with hemodynamic instability, such as cardiogenic shock, (6) patients with other comorbidities, (7) review/meta-analysis, and (8) articles on medical bioinformatics.

Independently, according to the inclusion and exclusion criteria, included data were extracted by two authors. MicroRNA, lncRNA, and circRNA are described as diagnostic and prognostic biomarkers for ACS. Moreover, microRNAs as diagnostic biomarkers for ACS are divided as increased expression, decreased expression, and contradictory expression.

## 3. Results and Discussion

### 3.1. MicroRNAs as Diagnostic and Prognostic Biomarkers for ACS

#### 3.1.1. MicroRNAs as Diagnostic Biomarkers for ACS

MicroRNAs (miRNAs) as diagnostic markers are divided into three types: miRNAs of increased expression in ACS, miRNAs of decreased expression in ACS, and miRNAs of contradictory expression in ACS. Moreover, we described these miRNAs of increased expression in ACS based on miRNA family (Table 1).

*(1) miRs of Increased Expression in ACS*.

- *miR-1*. The expression level of miR-1 in peripheral blood (plasma or serum) of AMI patients (including STEMI and NSTEMI patients) was significantly higher than that of healthy controls or patients with unstable angina [9–14], peaked at the same time as cTnI, and returned to baseline level 5 days after onset [13]. miR-1 in peripheral blood has a high diagnostic efficiency for AMI, and the area under the ROC curve is about 0.8 [9, 14], but it is not better than cTnT [11]. In addition, the expression level of mir-1 in peripheral blood of ACS patients was higher than that of non-ACS patients, and the expression level of miR-1 in UA patients was significantly higher than that of NSTEMI [15]. Although the expression level of miR-1 in the peripheral blood plasma or serum of AMI patients was increased, the expression level of miR-1 in the circulating monocytes of STEMI patients was significantly decreased [16]. The reason for this difference may be due to the cellular source of miR-1. The expression level of myocardial source is high, while the expression level of vascular source is low [17].
- *miR-133/133-a/133 b*. The expression level of miR-133 in the peripheral blood of patients with AMI increased significantly [18, 19], and returned to the normal level after 7 days [19], with high diagnostic efficiency for AMI and AUC of about 0.90 [18, 19]. Moreover, the expression level of miR-133a in the peripheral blood was significantly increased in ACS patients (including both AMI and UA) [12, 20–25]. The level of miR-133a in the peripheral blood began to increase 2 hours after the onset of symptoms [20] and peaked at about 21 hours [21], which was closely related to the severity of coronary artery lesions. The AUC for diagnosis of AMI or CHD was about 0.90 [21, 26]. In addition, the expression level of miR-133a/133b in peripheral blood of ACS patients with complete target vessel occlusion was significantly higher than that of patients with incomplete target vessel occlusion [23]. The level of miR-133a/133b could be used to identify whether the target vessel in ACS patients was totally occluded or not, so as to guide further treatment in a timely manner.
- *miR-208/208a/208b/208b-3p*. The expression of miR-208 in the peripheral blood of AMI patients increased [9, 10], with the AUC of 0.72 [27], but the expression level was lower than that of patients with angina pectoris [10]. miR-208a cannot be detected in plasma of healthy people or non-AMI patients, but it can be detected within 4 hours after onset of AMI [28], which is of great significance for early diagnosis of AMI. Compared with healthy controls [29, 30] and patients with unstable angina [12, 30], the circulating miR-208b level of AMI patients was significantly increased, the AUC value was less than 0.85 [31, 32], and the diagnostic efficiency was lower than cTnT [29, 31, 32].
- *miR-499/miR-499-5p*. The expression of circulating miR-499 was significantly increased in patients with ACS (including AMI and UA) [9, 10, 15, 24, 29, 31, 33–36], the AUC of AMI was 0.88 [9], the AUC of UA and NSTEMI was 0.98 and 0.97, respectively [34], and the diagnostic efficiency of ACS within 3 hours of onset was AUC of 0.89 [34]. In addition, the level of miR-499-5p was high in peripheral blood of patients with ACS [13, 37, 38], especially higher in NSTEMI patients [38], peaked later than cTnI, and returned to normal within 5 days [13]. AUC of AMI was 0.79 [32], while AUC of NSTEMI was 0.86 [38].
- *miR-21*. Circulating miR-21 in patients with ACS was significantly higher than that in patients with stable coronary heart disease/non-ACS [15, 39, 40], and it was also found that miR-21 was associated with visfatin [39] and mmp-9 (mmp-9) [40], which may participate in the pathogenesis of ACS by jointly promoting inflammatory response [39]. When miR-21 was combined with miR-1 and miR-499, the diagnostic efficiency was significantly improved (AUC 0.94), which was superior to the hypersensitive troponin T (AUC 0.89) [15]. Compared with angina pectoris or healthy control group, the expression level of miR-21 in plasma of AMI patients was significantly increased, and the increased level was related to CK-MB and cTnI. Further ROC analysis suggested that miR-21 had similar diagnostic efficiency with CK-MB and cTnI [41]. In addition, the expression level of circulating miR-21 was significantly higher in patients with unstable angina than in the control group, suggesting that circulating miR-21 could be used as a biomarker for unstable angina [42]
- *miR-134*. The expression level of circulating miR-134 was significantly increased in AMI patients [10, 43], and the level of circulating miR-134 in STEMI patients with complete target vessel occlusion was higher than that in NSTEMI patients with incomplete target vessel occlusion (AUC: 0.725, *p* = 0.002) [23]. miR-134 expression was higher in patients with unstable angina than in patients with stable angina [44].
- *miR-221-3p*. The expression of circulating miR-221-3p in AMI increased, and the diagnostic efficiency AUC value was 0.881 [45], which was also of certain significance for the identification of STEMI and NSTEMI [46].
- *miR-328*. The expression of miR-328 in both plasma and whole blood of AMI patients was significantly increased [19, 43], returned to normal after 7 days, and was not affected by arrhythmia. The AUC of AMI diagnosed by miR-328 in plasma and whole blood was 0.702 and 0.872, respectively [19].
- *miR486/miR-486-3p*. The expression level of serum mir-486-3p increased at the time of AMI, and the AUC of AMI diagnosed by the ratio of mir-486-3p/mir-191-5p was 0.863 [47]. Further studies have shown that circulating mir-486-3p levels can differentiate STEMI patients (at three months after onset) from patients with stable ischemic heart disease [48]. The level of miR-486 was also significantly higher in AMI patients than in healthy controls. When combined with miR-150, the AUC value was 0.771 in discriminating AMI patients from healthy controls and the AUC value was 0.845 in discriminating NSTEMI patients from healthy controls [49].
- *miR-124/miR-124-3p*. The expression of miR-124 in the peripheral blood of patients with AMI was significantly increased, peaked at 6 hours after onset, and the diagnosed AUC value was 0.86 [50]. Moreover, the expression of circulating miR-124 in AMI patients with complete target vessel occlusion was significantly higher than that in AMI patients with incomplete target vessel occlusion [23]. Circulating miR-124-3p expression was also significantly increased in ACS patients [37].
- *miR-19a, miR-19b, and miR-19b-3p*. Circulating miR-19a, miR-19b, and miR-19b-3p expression levels in the AMI group were higher than those in controls without AMI [51–53]. Moreover, plasma miR-19b-3p level reached peak expression immediately after admission [52]. The AUC value for miR-19b was 0.74 [51].
- *miR-30a, miR-30d, and miR-30d-5p*. Expression level of circulating miR-30d-5p was higher in patients with ACS compared with the healthy controls [54]. The AUC value of miR-30d-5p for ACS was high (0.915). Circulating miR-30a in AMI patients and circulating miR-30d in STEMI patients were highly expressed [55, 56]. When combined miR30a, miR-195, and let-7b, the AUC value for AMI was 0.93 at 8 h after onset and 0.92 at 12 h after onset.
- *miR-150 and miR-150-3p*. The levels of miR-150 and miR-150-3p were significantly higher in AMI patients than in healthy controls [47, 49]. The AUC value was 0.863 when using the ratio of miR-486-3p and miR-191-5p.
- *miR-181a and miR-181c*. Expression levels of circulating miR-181a and miR-181c^∗^ were higher in AMI patients [57, 58]. The AUC value of miR181-a for AMI was 0.834.
- *miR-25 and miR-25-3p*. The expression level of miR-25 in the plasma particles isolated from patients with unstable angina was significantly higher than those in the control group [42]. In addition, the expression level of miR-25-3p in peripheral blood plasma, platelets, and monocytes of STEMI patients was significantly higher than that of NSTEMI patients [45].
- *Other microRNAs*. The expression of circulating miR-195, miR-146a, miR-186-5p, miR-17-5p, miR-126-5p, miR-145-3p, miR-223, miR-483-5p, miR-26a-1, miR-199a-1, miR-1291, miR-663b, and miR-497 in AMI patients is significantly higher than that in the control group [16, 18, 51, 52, 55, 59–62]. The expression of miR-143 (monocytes) in STEMI patients was significantly higher than that in healthy controls [16]. The expression level of circulating miR-183-5p, miR-34a-5p, miR-323-3p, miR-652, miR-27b, miR-941, miR-122, miR-140-3p, miR-720, miR-2861, and miR-3149 in patients with ACS was significantly higher than those in patients without ACS or coronary heart disease [27, 37, 63–65]. The expression of circulating miR-3162-3p, miR-198, and miR-370 in UA patients was significantly higher than that in SA patients [44, 66], and the diagnosed AUC value of miR-3162-3p was 0.79. When combined with miR-3162-3p, miR-1225-5p, and miR-1207-5p, the AUC value was increased to 0.91 [67]. In addition, the expression level of miR-106b, miR-590-5p, miR-126^∗^, and miR-451 in the plasma particles isolated from patients with unstable angina was significantly higher than those in the control group [67].

*(2) miRs of Decreased Expression in ACS*. Plasma miR-126 expression level in patients with coronary heart disease (CHD) was significantly lower than that in the control group, and the trend of miR-126 expression level in coronary heart disease (CHD) was AMI < UAP < SAP [68]. The platelet-derived miR-126 level of STEMI patients was also significantly lower than that of the control group and was negatively correlated with plasma troponin I (*r* = −0.556, *p* = 0.011) [69]. The expression levels of circulating miR-126-3p, miR-26a-5p, miR-191-5p, miR-99a, miR-519e-5p, miR-1915, miR-320b, and miR-125b in AMI patients decreased significantly [47, 58, 70–72]. Among them, miR-99a is correlated with AMI severity [70]. The expression level of miR-519e-5p in patients with ischemic stroke and pulmonary embolism was increased, which was different from that in patients with AMI [71]. Decreased levels of miR-320b and miR-125b are associated with increased occurrence of AMI [72]. The expression levels of circulating miR-134-5p, miR-15a-5p, and let-7i-5p in STEMI patients were significantly lower than those in the control group [63]. In addition, compared with patients with stable angina, the expression levels of circulating miR-1202, miR-1207-5p, and miR-1225-5p in patients with unstable angina were decreased, and the latter two combined with miR-3162-3p could increase the diagnostic AUC value of elderly patients with unstable angina to 0.91 [67].

*(3) miRs of Contradictory Expression in ACS*. Although the expression level of miR-1 in the peripheral blood plasma or serum of AMI patients was increased, the expression level of miR-1 in the circulating monocytes of STEMI patients was significantly decreased [16]. The expression of circulating let-7b was decreased in AMI patients [55], but it was significantly upregulated in CD34+ progenitor cells mobilized by STEMI patients [73]. Circulating miR-92a expression was increased in patients with AMI or unstable angina [42, 74, 75], whereas miR-92a was decreased in peripheral blood monocytes of STEMI patients [16]. Compared with healthy controls, the expression of circulating miR-134-5p in STEMI patients was significantly downregulated [63]. However, the expression level of circulating miR-134-5p in patients with ACS or early AMI was significantly increased [37, 52]. The expression of miR-22-5p in plasma of patients with AMI was significantly decreased [76], but the expression of miR-22-5p in serum was significantly increased [77]. The expression of circulating miR-423-5p was downregulated within 24 hours in AMI patients [78], but increased within 6 hours in STEMI, unstable angina, and even AMI patients [26, 56, 79]. The expression of circulating miR-122 decreased in STEMI patients [13], but increased in ACS patients [65]. In addition, the expression of circulating miR-214 decreased in AMI patients, and the coronary lesion plaque is larger with the lower level [80]. However, circulating miR-214 expression was increased in CHD patients including AMI and angina [81]. The reason for the contradictory expression of the same miR probably is the different cell source that the expression level of myocardial source is high, while the expression level of vascular source is low [17]. It is also possibly related to different samples, different storage methods, and different reference genes.

#### 3.1.2. MicroRNAs as a Prognostic Marker for ACS

*(1) Cardiovascular Death/all-Cause Death*. Elevated circulating levels of miR-197, miR-223, miR-133a, and miR-208b were strong predictors of the risk of cardiovascular death in ACS patients, with the former two miR risk ratios of 2.24 (*p* = 0.006) and 4.94 (*p* = 0.012), respectively [12, 82]. Circulating miR-132 (hazard ratio 2.85, *p* = 0.022), miR-140-3p (hazard ratio 2.88, *p* = 0.022), and miR-210 (hazard ratio 3.10, *p* = 0.039) reliably predicted the risk of cardiovascular death in patients with ACS four years after discharge [83]. Circulating miR-208b, miR-34a, and miR-499-5p were significantly associated with increased risk of death or heart failure in AMI patients [32, 84], especially in patients who died within 30 days after AMI [31]. The longer the survival time is, the lower the expression level of circulating miR-208b is [85]. In addition, elevated circulating miR-328 and miR-134 levels are closely associated with a high risk of death or heart failure within 6 months of AMI [43]. Circulating miR-155, miR-380^∗^, and miR-499-5p were significantly increased in patients with cardiogenic death within 1 year after AMI/NSTEMI [86, 87].

*(2) Impaired Left Ventricular Function/Left Ventricular Remodeling/Heart Failure*. Baseline levels of circulating miR-652 were significantly associated with rehospitalization of ACS patients for heart failure and further improved risk stratification when combined with NT-proBNP and LVEF [27]. For patients with anterior wall AMI, low levels of circulating miR-150/miR-101 and high levels of circulating miR-16/miR-27a indicate a high risk of impaired left ventricular systolic function [88]. The expression levels of circulating miR-208b (OR 17.91, *p* = 0.003) and miR-34a (OR 4.18, *p* = 0.012) were increased in patients with AMI complicated with left ventricular remodeling, and their expression levels were associated with an increased risk of heart failure [30, 84]. Elevated circulating miR-328 and miR-134 levels in AMI patients are closely associated with a high risk of heart failure within 6 months [43]. Serum levels of miR-192, miR-194, and miR-34a related to p53 response were increased in patients with newly diagnosed heart failure within 1 year after AMI onset [89]. Low expression of circulating miR-145 was significantly correlated with increased serum B-type natriuretic peptide levels, increased troponin T levels, and decreased ejection fraction (all *p* < 0.0001). Multivariate linear regression analysis showed that AMI and heart failure were independently correlated with low expression levels of miR-145 [90]. Circulating miR-150 expression decreased in AMI patients with increased left ventricular end-diastolic volume, suggesting that low levels of circulating miR-150 are associated with left ventricular remodeling [91]. Moreover, serum miR-150 was significantly lower in patients with post-AMI heart failure than that in patients without AMI heart failure, and its level was significantly correlated with ejection fraction 1 year after AMI. The AUC value of post-AMI heart failure was 0.764, which was an independent predictor of heart failure after AMI [92]. Circulating miR-1 and miR-29b levels were correlated with changes in infarct volume of MRI indicators, and miR-29b levels were closely correlated with changes in left ventricular end-diastolic volume of MRI indicators [93], so increased circulating miR-1 levels could also predict the occurrence of heart failure after AMI [94].

*(3) Acute Renal Injury after AMI*. miR-24-3p, miR-23a-3p, and miR-145-5p can detect AMI early and may be involved in the pathogenesis of renal injury and fibrosis after AMI [95].

*(4) Predict the Risk of Future AMI*. Event-free survival of STEMI patients was significantly worse than that of patients with a higher miR-122-5p/133b ratio, with an almost 9-fold higher risk of death or myocardial infarction and a 4-fold higher risk of adverse cardiovascular events [96].

*(5) Correlated with the Severity of Coronary Lesions*. The expression level of circulating miR-155 in patients with multiple coronary artery lesions was significantly lower than that in patients with single or no coronary artery lesions, suggesting that the level of circulating miR-155 was negatively correlated with the severity of coronary artery lesions and various risk factors [97]. In addition, the expression of circulating miR-214 was decreased in AMI patients, and the lower the level is, the greater the plaque load in coronary arteries is [80].

*(6) Major Adverse Cardiovascular Events*. In patients with ACS, circulating miR-208b-3p (RR 1.225), miR-34a-5p (RR 0.963), and miR-499-5p (RR 0.077) were independently correlated with cardiovascular disease, myocardial infarction, and ischemic event risk, respectively, while miR-133b (RR 1.009) was only correlated with cardiovascular event risk [37]. Increased levels of circulating miR-133a and mirR208b or increased miR-122-5p/133b in AMI/STEMI patients were associated with a higher incidence of major adverse cardiovascular events [20, 30, 96]. Among them, increased levels of miR-122-5p/133b were associated with almost 9 times higher risk of death or myocardial infarction and 4 times higher risk of adverse cardiovascular events [96]. Circulating miR-125b-5p levels were closely correlated with cardiovascular events in AMI patients at 6 months [54]. In addition, circulating miR-145 was an independent and significant predictor of cardiac events (including cardiogenic death or worsening heart failure requiring hospitalization) within 1 year after AMI (risk ratio of 7.174, *p* < 0.0001) [98].

### 3.2. Long Noncoding RNAs as Diagnostic and Prognostic Markers for ACS

#### 3.2.1. Diagnosis for Recurrent AMI

The earliest study on lncRNA as a marker in ACS patients began in 2014. A total of 414 patients with acute myocardial infarction and 86 healthy volunteers were enrolled in this study to detect the expression of 5 lncRNAs in peripheral blood [99]. The results showed that the expression of 4 lncRNAs was significantly different between the two groups. Among them, hypoxia-inducible factor 1A antisense RNA 2, KCNQ1OT1, and metastasis-associated lung adenocarcinoma transcript 1 were significantly increased in circulation in patients with acute myocardial infarction (*p* < 0.01).

After that, it was found that Myosin Heavy Chain Associated RNA (MHRT) [100], CDR1AS (Cdr1 antisense) [101] LOC145474, LOC100129518, BRE-AS1, MIR22HG, MIR3945HG, ATP2B1-AS1, CATIP-AS1, LINC00528 [102], and ENST00000444488.1 [103] were significantly increased in the peripheral blood of patients with acute myocardial infarction, compared with healthy control group or nonacute myocardial infarction group. It can be used as a diagnostic marker for acute myocardial infarction. lncRNA MHRT can inhibit apoptosis and protect ischemic myocardium [100]. The AUC value of CDR1AS in the diagnosis of acute myocardial infarction was 0.671, which was an independent predictor of acute myocardial infarction [101]. When 8 upregulated lncRNAs (LOC145474, LOC100129518, BRE-AS1, MIR22HG, MIR3945HG, atp2b1-as1, catip-as1, and LINC00528) were combined with 3 downregulated lncRNAs (wdr86-as1, a2m-as1 and LINC00612), the AUC value of these lncRNAs in diagnosing acute myocardial infarction was 0.701-0.955. Further functional enrichment analysis showed that these 11 lncRNAs were, respectively, related to inflammation and immunity [102]. Circulating ENST00000444488.1 was more than 15 times higher in AMI patients than in non-AMI patients, contributed to the diagnosis of acute myocardial infarction, and it was involved in the regulation of inflammation-related genes in AMI patients [103].

In addition to the increased expression level of circulating lncRNAs in patients with acute coronary syndrome, some expressions were also decreased. ANRIL (*p* = 0.003) [99], circulating ZFAS1 (any Zinc finger antisense 1) established (*p* < 0.0001) [101], lncRNA urothelial carcinoma associated factor 1 (urothelial carcinoma-associated 1, UCA1) [104], WDR86-AS1, A2M-AS1, LINC00612 [102], lncRNA HOTAIR [105], uc010yfd.1 [103], lncRNAs ENST00000416860.2, ENST00000421157.1, and TCONS_00025701 [106] were significantly decreased in the peripheral blood of patients with acute myocardial infarction and could also be used as circulating markers of acute myocardial infarction. Among them, the AUC value diagnosed by cyclic ZFAS1 was 0.664. UCA1 expression was decreased in plasma at the early stage of acute myocardial infarction and increased after 3 days after myocardial infarction, contrary to circulating miR-1 expression [104]. Overexpression of HOTAIR can inhibit hypoxia-induced myocardial apoptosis, and the myocardial protective effect of HOTAIR is partly based on negative regulation of miR-1 [105]. Studies of Uyghur people in China showed that lncRNA ENST00000416860.2, ENST00000421157.1, and TCONS_00025701 in peripheral blood of AMI patients decreased significantly compared with healthy people. It is worth noting that TCONS_00025701 decreased most significantly. Functional enrichment analysis indicated that these lncRNAs were associated with apoptosis and P53 [106].

#### 3.2.2. Used to Identify AMI and UA

Seven lncRNAs (RP11 68i3.11, AC068831.6, RP11 133l14.5, PA X8 AS1, RP11 259k15.2, RP11 2 03m5.8, and LINC01254) were significantly abnormal in the expression of ACS patients and could be used to differentiate acute myocardial infarction from unstable angina pectoris, with diagnostic accuracy, sensitivity, and specificity of 90.38%, 100%, and 68.75%, respectively. The AUC value of differential diagnosis can reach 0.976 [107].

#### 3.2.3. Used to Identify STEMI and NSTEMI

In comparison with NSTEMI, lncRNA ANRIL (*p* < 0.001), KCNQ1OT1 (*p* < 0.001), myocardial infarction-associated transcript (*p* < 0.001), and metastatic adenocarcinoma transcript 1 (*p* = 0.005) in the circulation of STEMI patients were significantly decreased. The expression profile of this group of lncRNA can be used to identify two different pathological mechanisms of myocardial infarction [99]. According to the latest studies, 6 lncRNAs (LNC_001526, LNC_000461, LNC_002674, ENST00000508020.2, ENST00000635852.1, and LNC_001265) were significantly higher in the peripheral blood of STEMI patients than those of NSTEMI patients, which could be used to identify STEMI and NSTEMI in AMI. ENST00000508020.2, LNC_001265, LNC_001526, and LNC_002674 had better sensitivity and specificity. Further enrichment analysis indicated that these lncRNAs were associated with cell adhesion, calcium homeostasis, complement receptor-mediated signaling pathways, and immune system processes [108].

#### 3.2.4. As a Prognostic Marker of Heart Failure

Long noncoding RNA ANRIL, KCNQ1OT1, myocardial infarction-related transcripts, and metastatic lung adenocarcinoma transcripts 1 can significantly predict impaired left ventricular cardiac function (ejection fraction ≤40%) at 4 months [99].

### 3.3. circRNAs as Diagnostic and Prognostic Markers for ACS

circRNAs have only been gradually paid attention to as cyclic markers of ACS in recent years, so there are few relevant studies. Current studies have shown that a type of myocardial infarction-associated circular RNA (MICRA) cannot be detected in the plasma or serum of AMI patients and is mainly expressed in peripheral blood cells [109, 110]. Compared with healthy controls, MICRA expression in peripheral blood cells of AMI patients was significantly decreased (*p* = 0.003) [110]. Multivariate analysis showed that MICRA was a strong predictor of left ventricular dysfunction (OR 0.53, 95% CI: 0.29-0.97) [110]. After AMI, MICRA expression level in peripheral blood cells of patients with significantly decreased ejection fraction was lower than that of patients with retained ejection fraction or slightly decreased ejection fraction [109]. Moreover, the MICRA can predict the left ventricular function (ejection fraction) 4 months after AMI [109, 110].

## 4. Conclusion and Prospect

At present, studies on ncRNA as a diagnostic and prognostic marker of ACS mainly focus on micro RNA, while there are relatively few studies on lncRNA and circRNA. Moreover, researches on microRNAs mainly focus on acute myocardial infarction, while researches on unstable angina or ACS are relatively seldom. Currently, due to the application of troponin, especially hypersensitive troponin, the diagnostic efficiency of acute myocardial infarction has been greatly improved with high sensitivity and specificity. Although some microRNAs have been successively found in the peripheral blood of patients with acute myocardial infarction as diagnostic markers, compared with the gold standard (troponin) currently used in clinical practice, these microRNAs cannot show better diagnostic efficiency. Therefore, further studies on ncRNAs, especially lncRNAs and circRNAs, are needed in the future. For research on miRNAs, more attention should be paid to the markers of UA in ACS, and the search for miRNAs should be more specific and sensitive than troponin.

## Figures and Tables

**Table 1:** miRs of increased expression in ACS.

Gene/gene family name	Relevant disease	Samples
miR-1	ACS	Peripheral blood
miR-133/133-a/133 b	ACS	Peripheral blood
miR-208/208a/208b/208b-3p	AMI	Peripheral blood/plasma
miR-499/miR-499-5p	ACS	Peripheral blood
miR-21	ACS/AMI	Plasma
miR-134	AMI/STEMI/NSTEMI/UA	Serum/plasma/peripheral blood mononuclear cell
miR-221-3p	AMI	Plasma, platelets, and leukocytes
miR-328	AMI	Plasma/whole blood
miR-486/miR-486-3p	AMI	Plasma/serum
miR-124/miR-124-3p	AMI/ACS	Peripheral blood/serum
miR-19a/miR-19b/miR-19b-3p	AMI	Plasma/peripheral blood
miR-30a/miR-30d/miR-30d-5p	AMI/STEMI/ACS	Plasma/peripheral blood
miR-150/miR-150-3p	AMI	Serum
miR-181a and miR-181c	AMI	Plasma/peripheral whole blood
miR-25 and miR-25-3p	UA/STEMI/NSTEMI	Plasma
Other microRNAs (miR-17-5p/26a-1/27b/34a-5p/106b/122/126^∗^/126-5p/140-3p/143/145-3p/146a/183-5p/186-5p/195/198/199a-1/223/323-3p/370/451/483-5p/497/590-5p/652/663b/720/941/1291/2861/3149/3162-3p)	AMI/STEMI/ACS/UA	Particles/plasma/monocytes
